# Comprehension of Subject and Object Relative Clauses in a Trilingual Acquisition Context

**DOI:** 10.3389/fpsyg.2017.01641

**Published:** 2017-10-06

**Authors:** Angel Chan, Si Chen, Stephen Matthews, Virginia Yip

**Affiliations:** ^1^Department of Chinese and Bilingual Studies, Hong Kong Polytechnic University, Hong Kong, China; ^2^Speech Therapy Unit, Hong Kong Polytechnic University, Hong Kong, China; ^3^Hong Kong Polytechnic University-Peking University Research Centre on Chinese Linguistics, Hong Kong, China; ^4^Childhood Bilingualism Research Centre, The Chinese University of Hong Kong, Hong Kong, China; ^5^Department of Linguistics, The University of Hong Kong, Hong Kong, China

**Keywords:** child second and third language acquisition, cross-linguistic influence, input conditions, structural overlaps, typological distance, Cantonese, Mandarin, English

## Abstract

Chinese relative clauses (RCs) have word order properties that are distinctly rare across languages of the world; such properties provide a good testing ground to tease apart predictions regarding the relative complexity of subject and object RCs in acquisition and processing. This study considers these special word order properties in a multilingual acquisition context, examining how Cantonese(L1)-English(L2)-Mandarin(L3) trilingual children process RCs in two Chinese languages differing in exposure conditions. Studying in an English immersion international school, these trilinguals are also under intensive exposure to English. Comparisons of the trilinguals with their monolingual counterparts are made with a focus on the directionality of cross-linguistic influence. The study considers how various factors such as language exposure, structural overlaps in the target languages, typological distance, and language dominance can account for the linguistic abilities and vulnerabilities exhibited by a group of children in a trilingual acquisition context. Twenty-one trilingual 5- to 6-year-olds completed tests of subject- and object- RC comprehension in all three languages. Twenty-four age-matched Cantonese monolinguals and 24 age-matched Mandarin monolinguals served as comparison groups. Despite limited exposure to Mandarin, the trilinguals performed comparable to the monolinguals. Their Cantonese performance uniquely predicts their Mandarin performance, suggesting positive transfer from L1 Cantonese to L3 Mandarin. In Cantonese, however, despite extensive exposure from birth, the trilinguals comprehended object RCs significantly worse than the monolinguals. Error analyses suggested an English-based head-initial analysis, implying negative transfer from L2 English to L1 Cantonese. Overall, we identified a specific case of bi-directional influence between the first and second/third languages. The trilinguals experience facilitation in processing Mandarin RCs, because parallels and overlaps in both form and function provide a transparent basis for positive transfer from L1 Cantonese to L3 Mandarin. On the other hand, they experience more difficulty in processing object RCs in Cantonese compared to their monolingual peers, because structural overlaps with competing structures from English plus intensive exposure to English lead to negative transfer from L2 English to L1 Cantonese. The findings provide further evidence that head noun assignment in object RCs is especially vulnerable in multilingual Cantonese children when they are under intensive exposure to English.

## Introduction

Relative clauses have been intensely investigated in language typology, acquisition and processing for decades. Chinese relative clauses have word order properties that are otherwise rare across the languages of the world. Given these special word order properties, Chinese languages are important in debates regarding acquisition and processing of RCs because they allow researchers to tease apart predictions regarding the relative complexity of subject vs. object RCs. Moreover, relative clauses in Cantonese and Mandarin differ enough for there to be language-specific effects on acquisition (Chan et al., 2011).

In this study, we take on a new perspective by considering these special word order properties in a multilingual acquisition context, examining how Cantonese(L1)-English(L2)-Mandarin (L3) trilingual children process relative clauses in two Chinese languages acquired under different exposure conditions. The trilinguals come from Hong Kong middle class families where they are exposed to Cantonese as first language in the family and community from birth, and to Mandarin at school for only 200 min per week. Being educated in an English immersion international school, the trilinguals acquire these two Chinese languages under intensive exposure to English as a second language. Comparisons of the trilingual children with their monolingual counterparts are made with a focus on the directionality of cross-linguistic influence. The study considers language exposure, structural overlaps in the target languages, typological distance, perceived language distance, and language dominance as factors leading multilingual children to experience facilitation in one instance and competing analyses in another, when processing relative clauses (Chan et al., 2011; Kidd et al., 2015).

The study is novel in a number of ways. First, it is the first experimental study of relative clause comprehension in Cantonese-English-Mandarin trilingual children. Second, we demonstrate a specific instance of bi-directional influence between first and second/third languages in this syntactic domain in a trilingual acquisition context. In particular, we argue for forward positive transfer from L1 Cantonese to L3 Mandarin and reverse negative transfer from L2 English to L1 Cantonese taking place within a single grammatical domain in this group of trilinguals. L2-to-L1 transfer has been documented in a number of studies involving a variety of language pairs, although to date, a majority of the studies feature adult second language acquisition in a largely European language context (e.g., Cook, 2003; Dussias and Sagarra, 2007; Morett and MacWhinney, 2013; but see also Liu et al., 1992; Su, 2001 involving Mandarin and English in adult second language acquisition). Third, this study features the acquisition of Chinese under strong English influence, a phenomenon that is increasingly common among not only children in Hong Kong who are being educated in an international school curriculum, but also relevant to a significant number of Chinese immigrant or adopted children around the world who are typically exposed to a Chinese language at home and grow up in an English-speaking country where they acquire the English language of the speech community simultaneously or successively. These multilingual children form a significant emerging group facing the challenge of preserving Chinese as their heritage language and acquiring English as the mainstream language of the community and/or school in which they grow up.

### Relative clauses in Cantonese, Mandarin, and English

While English and Chinese share the basic word order SVO, they differ in that relative clauses (RCs) are consistently placed before the head noun in Chinese. See (1) and (2) for an example of a subject RC, and (3) and (4) for an example of an object RC in Cantonese and Mandarin respectively. In fact, pre-nominal RCs plus SVO main clause word order is a rare combination cross-linguistically (Dryer, 2013).

**Cantonese subject RC** (CL: classifier; SFP: sentence final particle):

(1) [**_RC____i_**  錫     公雞][_head noun_   嗰     隻      老鼠_i_]
                    sek3  gung1gai1       go2   zek3  lou5syu2
                    kiss   chicken           that   CL    mouse
                   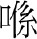
邊度           呀?
                   hai2   bin1dou6        aa3
                   is       where             SFP
                   “Where's the mouse that kisses the chicken?”

**Mandarin subject RC:**

(2) [**_RC____i_**  親      公雞]      的 [_head noun_  老鼠_i_]    在   哪裡?
                   qing   gongji    de               laoshu  zai  nali
                   kiss   chicken  de               mouse  is    where
                   “Where's the mouse that kisses the chicken?”

**Cantonese object RC:**

(3) [**_RC_** 老鼠          錫___ _j_] [_head noun_   嗰     隻       公雞_j_]
            lou5syu2   sek3                      go2  zek3   gung1gai1
            mouse       kiss                       that  CL     chicken
            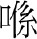       邊度              呀?
            hai2           bin1dou6               aa3
            is               where                    SFP
            “Where's the chicken that the mouse kisses?”

**Mandarin object RC:**

(4) [**_RC_**   老鼠       親___ _j_]   的 [_head noun_ 公雞_j_]    在     哪 裡?
              laoshu    qing        de              gongji   zai    nali
              mouse    kiss        de              chicken is      where
              “Where's the chicken that the mouse kisses?”

**Figure 1:**
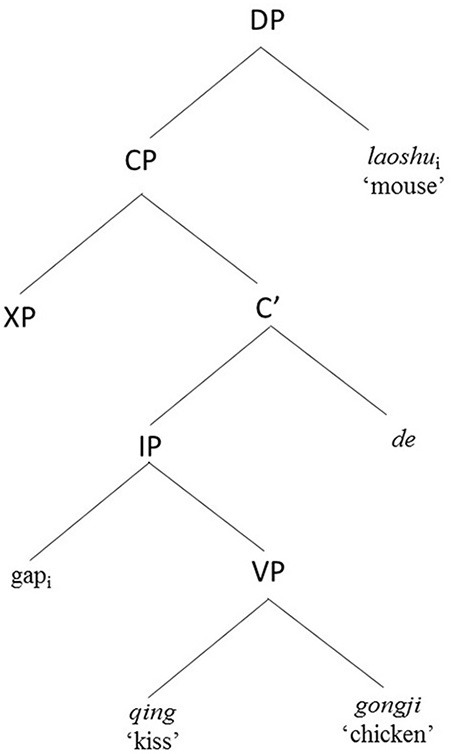
Hierarchical structure of a Mandarin subject RC.

**Figure 2:**
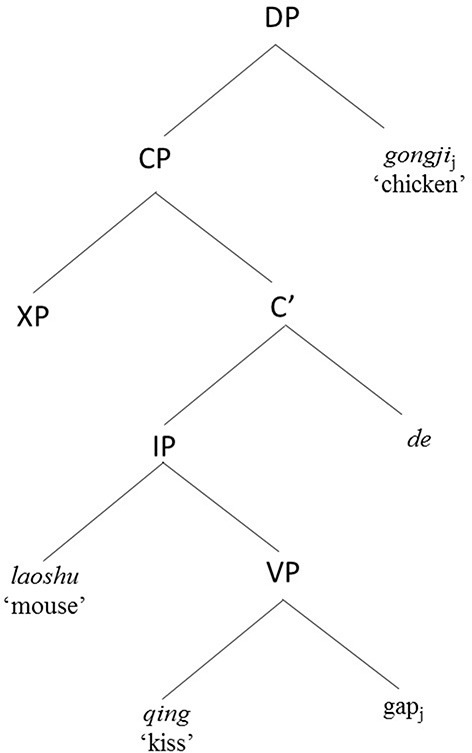
Hierarchical structure of a Mandarin object RC.

As illustrated by examples (1) to (4), placing the RC before the head noun results in Chinese subject RCs having non-canonical VOS word order and a longer linear distance between filler and gap, while Chinese object RCs match the canonical SVO word order and have a shorter linear filler-gap distance. These structural configurations result in competing processing demands described as follows. On the one hand, Chinese subject RCs are less costly to process due to general subject prominence based on functional notions such as topicality: given that relative clauses describe the referent of their head noun and a clause's subject constitutes the default topic, it is less effortful to construe a RC as being about its default topic (the subject) than to construe it as being about some other item (Keenan and Comrie, 1977; Kim and O'Grady, 2015). From a formalist perspective, Chinese subject RCs are also easier to process in terms of lack of structural intervention in a hierarchical structure (Hu et al., 2015a,b). Along the lines of the locality principle of Relativized Minimality (Rizzi, 1990), a local relation between X (the relative head noun in the case of RCs) and Y (the copy of the moved relative head noun in the gap position) cannot hold if there is an intervener, Z, which is of the same structural type as X, and can be a potential candidate for the relation. In Chinese subject RCs like Figure 1, there is no structural intervener between the relative head (*laoshu* “mouse”) and its copy in the gap position. However, in Chinese object RCs like Figure 2, the embedded subject (*laoshu* “mouse”) intervenes between the relative head (*gongji* “chicken”) and its copy in the gap position, and qualifies as a potential candidate for the local relation. This makes the correct computation of the local relation more complex to resolve for children when they process Chinese object RCs. On the other hand, Chinese subject RCs are also more costly in terms of having to resolve a longer linear relationship between the filler and the gap [compare the distance between the gap and the filler (i.e., head noun) “mouse” in (1) and (2) vs. the distance between the gap and the head noun “chicken” in (3) and (4)], and in terms of deviating from the canonical SVO word order (Bever, 1970; Gibson, 1998, 2000; Diessel and Tomasello, 2005). What makes Chinese RCs intriguing is that, unlike English, subject prominence or structural influences, and linear influences such as similarity to canonical SVO word order and shorter linear distance between filler and gap, are no longer confounded to favor subject over object RCs, but work in opposite directions to both favor and disfavor subject RC processing. In Chan et al. (2011) and subsequently in Kidd et al. (2015), we argued that the processing and acquisition of Chinese RCs bear on the theoretical themes of competition and variation (MacWhinney, 2005, 2012).

### Chinese relative clause processing and cross-linguistic influences in multilingual acquisition

We now turn to discussing why the processing of Chinese RCs is interesting in a multilingual acquisition context. We focus on cross-linguistic influence, where structural overlaps between languages have been identified as a pre-condition for transfer (Hulk and Müller, 2000; see section Current Study and Hypotheses for further elaborations). First, since Cantonese and Mandarin are typologically close, their RCs overlap both structurally and functionally. For instance, the Cantonese subject RC in (1) and the Mandarin subject RC in (2) are highly similar. Likewise, the Cantonese object RC in (3) and the Mandarin object RC in (4) are highly similar. It is therefore reasonable to expect that the structural and functional overlaps between Cantonese and Mandarin RCs could provide a transparent basis for positive transfer between these two Chinese languages when individuals learn these two languages in multilingual acquisition. By contrast, the second point relates to vulnerability to negative cross-linguistic influence in multilinguals, and this requires us to first highlight an important difference between Cantonese and Mandarin RCs. As mentioned, Cantonese object RCs and Mandarin object RCs are highly similar [compare (3) and (4)]; however, there is also an important difference between them in terms of degree of overlap with SVO main clauses. One important structural feature unique to Cantonese object classifier RCs [see (3)] is that they share an identical surface structure with a SVO main clause, and as such instantiate a complete structural overlap with SVO transitive main clauses. Compare the object classifier RC in (3) repeated below as (5) and a Cantonese SVO main clause in (6).

**Cantonese object classifier RC**

(5) [**_RC_**    老鼠         錫___ _j_] [_head noun_ 嗰     隻     公 雞_j_]
               lou5syu2  sek3                    go2  zek3 gung1gai1
               mouse      kiss                     that  CL   chicken
               “The chicken that the mouse kisses”

**Cantonese transitive SVO main clause**

(6) [**_MC_**  老 鼠          錫       嗰   隻      公 雞]
              lou5syu2    sek3   go2   zek3   gung1gai1
              mouse       kiss     that   CL     chicken
              “The mouse kisses the chicken”

Interestingly, a recent study by Lau (2016a) elicited native Cantonese adult speakers' production of object classifier RCs like (5) in one condition and their production of transitive SVO main clauses like (6) which were identical in surface form in another condition, and the acoustic analyses found no prosodic differences between examples like (5) vs. (6). The results suggested that adult native speakers of Hong Kong Cantonese do not use prosody to disambiguate surface identity in syntax between object classifier RCs and transitive main clauses. Note that this characteristic of surface identity is unique to Cantonese object classifier RCs, but not Mandarin object RCs, because Mandarin object RCs [see (7)] only resemble but are not identical in surface structure with SVO main clauses, due to the presence of the relative marker *de*. Compare the Mandarin object RC repeated in (7) and a Mandarin SVO main clause in (8).

**Mandarin object RC**

(7) [**_RC_**    老 鼠   親___ j]  的 [_head noun_  公雞_j_]
               laoshu  qing      de               gongji
               mouse  kiss      de               chicken
               “The chicken that the mouse kisses”

**Mandarin transitive SVO main clause**

(8) [**_MC_**  老鼠       親      公雞]
              laoshu   qing   gongji
              mouse   kiss   chicken
              “The mouse kisses the chicken”

This surface identity between object classifier RCs and SVO main clauses in Cantonese presents advantages and challenges in acquisition and processing. Merit-wise, in Chan et al. (2011), we argued that Cantonese object classifier RCs allow for and could be facilitated by an internally headed RC analysis. Specifically, object classifier RCs like (5) can be analyzed as internally headed RCs as (9):

(9) [_NP/S_   lou5syu2  sek3    go2    zek3  gung1gai1]
                 mouse     kiss     that    CL    chicken.
                 “The chicken that the mouse kisses”

Under the internally headed RC analysis, example (9) has the internal structure of a SVO clause, but behaves as a noun phrase (NP) in terms of its external syntax. The internally headed RC analysis is represented by the notation NP/S in (9) above, indicating that a constituent has externally the syntax of a NP but internally that of a clause (S). Here the internal structure is a SVO main clause, with the object, which is also the head noun, *in situ*. Hence the head “chicken” is internal to the RC. Internally headed RCs do not involve gaps or extraction, are structurally simpler, and therefore may be easier to process than externally headed RCs (see Jeon and Kim, 2007 for supportive evidence from Korean). This internally headed analysis is only possible for Cantonese object classifier RCs because it is only in this case where there is complete surface identity with simple main clauses and therefore ambiguity of analysis. Examples like (5), as such, are structurally ambiguous as they can be analyzed as head-final RCs (5) or internally headed RCs (9).

Moreover, Cantonese learners could make use of simple transitives to bootstrap onto Cantonese object RCs, especially of the classifier type, in production. On the other hand, we also acknowledged that their surface identity with SVO main clauses could cause problems in comprehension, notably by leading Cantonese object classifier RCs to be mis-parsed as SVO transitive main clauses (Lau, 2016b), due to structural ambiguity. The potential to be misled due to competing analyses in sentence parsing could become more complicated for multilingual children acquiring Cantonese RCs under heavy influence from English, especially when there are additional competing constructions due to structural overlaps between the children's languages. This brings us back to the second point about vulnerability to negative cross-linguistic influence in multilinguals. Specifically, parsing of Cantonese object classifier RCs could be especially challenging for these multilingual children, because the Cantonese object RCs not only overlap with SVO in Cantonese but also SVO transitive clauses and head-initial subject RCs in English. Compare (5) and (6) repeated below as (10) and (11), alongside the English transitive SVO main clause in (12) and the English subject RC in (13).

**Cantonese object classifier RC (head-final)**

(10) [**_RC_** 老鼠         錫___ _j_] [_headnoun_    嗰     隻      公雞_j_]
              lou5syu2  sek3                      go2  zek3  gung1gai1
              mouse kiss that CL chicken
              “The chicken that the mouse kisses”

**Cantonese transitive SVO main clause**

(11) [**_MC_** 老鼠           錫      嗰      隻       公雞]
               lou5syu2   sek3   go2   zek3   gung1gai1
               mouse kiss that CL chicken
               “The mouse kisses the chicken”

**English transitive SVO main clause**

(12) The mouse kisses the chicken

**English subject RC (head-initial)**

(13) [_headnoun_ **The mouse**_j_] [_RC_ that ___ _j_ kisses the chicken]

Overlap with English head-initial subject RCs (also SVO) may encourage a head-initial analysis here. In particular, when Cantonese (head-final) object classifier RCs lack an overt relative marker introducing the head noun of the RC, head noun assignment could be especially vulnerable to negative cross-linguistic influence from English.

In fact, cross-linguistic influences have been observed in our previous work on simultaneous Cantonese-English bilingual children. Yip and Matthews (2007a) analyzed naturalistic speech production and found that object relative clauses (the classifier type, such as (3) but often with an inanimate head noun and an animate subject NP in the RC) emerged earlier than or simultaneously with subject relative clauses [such as (1)] in the bilingual children's Cantonese; while in their English, Cantonese-based prenominal relatives emerged first, with object relatives (e.g., “*Where's [*_*NP*_ *you buy that one]”* meaning “where's the one you bought” (example 15 from Yip and Matthews, 2007b) followed by subject relatives (e.g., “*I want [*_*NP*_ *have ear that one]”* meaning “I want the one that has ears” (example 20 from Yip and Matthews, 2007a). On the other hand, in a comprehension experiment, Kidd et al. (2015) found that their bilingual children made more head noun errors than the monolinguals when comprehending Cantonese object RCs that are consistent with an English head-initial analysis, erroneously choosing the subject of the RC [the first noun of the complex noun phrase, i.e., the “mouse” in (10)] rather than the “chicken” in (10) as the head noun.

Looking broader beyond Chinese and English in the context of the current literature, we highlight the following observations. First, descriptions of cross-linguistic influence in trilingual acquisition have largely featured adult learners and English and European languages (e.g., Cenoz and Jessner, 2000; Cenoz et al., 2001). Studies featuring cross-linguistic interactions in trilingual children exist, but many of which are case studies featuring a few children (Hoffmann, 1985; Helot, 1988; Li, 2006 inter alia.). Experimental studies testing a group of trilingual children have been relatively few. Regarding cross-linguistic influences in trilingual children, the broad trends of investigation have been on reporting the observed code-switching and mixing patterns between languages (e.g., Stavans and Swisher, 2006; Edwards and Dewaele, 2007; Hoffmann and Stavans, 2007; Stavans and Muchnik, 2008) and how the prior languages affect the acquisition of a third language (e.g., Oksaar, 1977; Hoffmann, 1985; Flynn et al., 2004; Anastassiou and Andreou, 2014). For instance, Oksaar (1977) identified negative transfer of semantics of verbs from the two L1s Estonian and Swedish of a child to his L3 German. However, we know relatively little about how the latter acquired languages affect the prior acquired languages (so called “reverse” transfer) from the current literature on trilingual children. A notable exception is Kazzazi (2011), which approached cross-linguistic influence in 2 trilingual children from a cognitive perspective. This study found that the post-modifying order in the non-dominant language Farsi was transferred to the other two languages (German and English) because this order manifests the general cognitive tendency toward iconicity and transparency. Thus, far there has been very little research on childhood trilingualism which approaches the issue of cross-linguistic influence from the theoretical perspective of structural overlaps between languages. On the other hand, cross-linguistic transfer due to structural overlaps has been more intensively studied in the bilingualism literature (see Serratrice, 2013 for a review).

### Current study and hypotheses

As a follow-up to our previous works (Yip and Matthews, 2000, 2007a; Kidd et al., 2015), we extend our work on cross-linguistic influences by examining a new group of multilingual children. Unlike our previous work that investigated simultaneous Cantonese-English bilingual children in Hong Kong (Yip and Matthews, 2000, 2007a) and in Australia (Kidd et al., 2015), we target a group of Cantonese-English-Mandarin trilingual children that is unique and relevant to a significant number of children in Hong Kong studying in international schools/curriculums with an English immersion environment from an early age. These children acquire Cantonese as their family and first language, and also acquire English and Mandarin as second and third languages at school. Although English is not the community language of Hong Kong, these children's Chinese is under heavy influence from English because they are educated in an English immersion environment. Specifically, we tested how Cantonese-English-Mandarin trilingual children's comprehension of subject and object RCs was influenced by the structural overlaps between the three languages when the two Chinese languages are acquired under different exposure conditions. These patterns of overlaps and differences may raise new possibilities for interactions between the three developing linguistic systems in trilingual children.

The current study draws reference to a number of theoretical perspectives in bilingual and multilingual acquisition and the kinds of transfer these perspectives predict. In particular, we draw reference to Hulk and Müller's specific hypothesis related to cross-linguistic influence in childhood bilingualism research (Hulk and Müller, 2000; Müller and Hulk, 2001). In addition, we consider several factors that have been proposed to drive the directionality of cross-linguistic influences, namely, typological distance, psychotypology, and language dominance. Hulk and Müller's hypothesis and these factors will be introduced briefly below, which will contribute to the formulation of our hypotheses specific to the current study.

In Hulk and Müller's hypothesis, one necessary condition for cross-linguistic influence to occur is partial structural overlap between the two languages regarding the structure of interest. Their original hypothesis defined the structural overlap condition as such: “syntactic cross-linguistic influence occurs only if language A has a construction which may seem to allow more than one syntactic analysis and, at the same time, language B contains evidence for one of these two possible analyses. In other words there has to be a certain overlap of the two systems at the surface level” (Hulk and Müller, 2000, p. 228–229). According to this hypothesis, if a structure in language A is potentially ambiguous between more than one analysis, and that language B allows only one of the analysis, there will be unidirectional influence from language B to language A in that the overlapping analysis would be adopted by the bilinguals more often than by the monolinguals. Another potential factor affecting directionality of cross-linguistic influence is typological distance (or linguistic distance). It has been proposed to be a major factor in the choice of the source language regarding cross-linguistic influence in multilingual language acquisition (Cenoz, 2001). This perspective is supported by the observation that speakers tend to transfer more vocabulary items and structures from the language that is typologically closer to the target language. A related notion is the concept of psychotypology by Kellerman (1983), that is, the language that is “perceived” as typologically closer. The role of psychotypology has been demonstrated in the literature. For instance, learners of English and French whose first language is a non-Indo-European language would tend to transfer vocabulary and structures from other Indo-European languages they know rather than from their L1 (Ahukanna et al., 1981; Ringbom, 1987; Bartelt, 1989). In addition, language dominance is another factor that can predict cross-linguistic influence: the source language tends to be the more dominant language (Yip and Matthews, 2000, 2007b).

We have two hypotheses focusing on the two Chinese languages for the current study. First we hypothesize that these trilingual children would experience facilitation in comprehending RCs in their third language Mandarin even with limited exposure, due to positive influence from their first language Cantonese. In particular, we expect that positive transfer from Cantonese to Mandarin allows the trilingual children to comprehend Mandarin RCs above the level that would be expected based on their limited input (as reflected by their weak vocabulary knowledge in Mandarin). Here we take vocabulary score as a proxy variable for a child's language-specific experience, and therefore expect that the trilinguals' Mandarin vocabulary scores would be significantly lower than their age matched monolingual Mandarin peers. However, by contrast, we expect that the trilinguals would not score as much lower than their monolingual age peers in their Mandarin RC comprehension performance as in their vocabulary scores, and they might even perform comparable to their monolingual age peers. The first hypothesis is motivated by the typological close proximity between Cantonese and Mandarin, and their similar RC structures in particular [compare (1) and (2)], coupled with the fact that Cantonese is the more dominant language while Mandarin is the weaker language for the trilingual children under investigation.

Second, we hypothesize that these trilingual children would experience more difficulty in comprehending Cantonese object classifier RCs relative to their monolingual peers, especially in head noun assignment, due to negative influence from English and intensive exposure to English. We therefore expect that the trilinguals would make significantly more head noun errors than their monolingual peers when comprehending Cantonese object classifier RCs, with the error pattern consistent with an English-based head-initial analysis. This hypothesis is motivated by the consideration that Cantonese object classifier RCs are potentially ambiguous between more than one analysis as described in section Trilingual vs. Monolingual Mandarin above, and these Cantonese object classifier RCs overlap with subject RCs in English when the two languages are in contact in a multilingual child, while English RCs clearly allow only a head-initial analysis. As such, transfer from English to Cantonese is possible based on Hulk and Müller's hypothesis.

## Methods

### Participants

Sixty-nine (*N* = 69) children participated. Twenty-one (*N* = 21, 10 females) Cantonese(L1)-English(L2)-Mandarin(L3) trilingual children were recruited from an international English-immersion elementary school in Hong Kong. Twenty-four (*N* = 24, 11 females) predominantly monolingual Cantonese-speaking children in Hong Kong, and 24 (*N* = 24, 11 females) monolingual L1 Mandarin children in China, served as comparison groups for the two Chinese languages. The predominantly monolingual Cantonese children were born in Hong Kong, spoke Cantonese at home, and the primary language of instruction at school is Cantonese. The trilingual group was aged between 5;4 and 6;1 (*M*_age_ = 5;8, *SD* = 0;2). The comparison groups were matched by age for both Cantonese and Mandarin: the monolingual Cantonese group was aged between 5;4 and 6;4 (*M*_age_ = 5;11, *SD* = 0;3) and the monolingual Mandarin group was aged between 5;9 and 6;5 (*M*_age_ = 5;11, *SD* = 0;2). Our trilingual English data showed the subject over object RC advantage well-attested in English, so we did not test a monolingual English comparison group. Table 1 summarizes the participant information.

**Table 1:** Subject information.

	**Trilingual Cantonese-Mandarin-English**	**Monolingual Cantonese**	**Monolingual Mandarin**
N	21	24	24
Age Range	5;4–6;1	5;4–6;4	5;9–6;5
Mean age	5;8 (*SD* = 0;2)	5;11 (*SD* = 0;3)	5;11 (*SD* = 0;2)

The trilingual children come from Hong Kong middle class families with both parents being native speakers of Cantonese. They have been exposed to Cantonese in the family and community from birth. These children became regularly and intensively exposed to English when they entered kindergarten around the age of 3. At the time of testing, they were attending an international English-immersion primary school five and a half hours a day and 5 days a week, during which they also received regular but far less extensive exposure to Mandarin as a foreign language for 200 min per week. The children reported speaking both Cantonese and English at home.

#### Trilingual children's language proficiency

The Cantonese Receptive Vocabulary Test (CRVT; Cheung et al., 1997) was used to assess the children's receptive Cantonese vocabulary knowledge. This standardized test provides norms based on monolingual Cantonese children in Hong Kong aged 2;0–6;0, giving some objective measure of the children's proficiency in Cantonese^1^. For Cantonese, the trilinguals scored on average 60 out of a total of 65 items in the CRVT correct. The majority of the trilingual children scored comparably to their monolingual age peers in the normative sample of the test (age equivalent according to CRVT: *M*_age_ = 5;8, *SD* = 0;4, *Range* = 5;0–6;1), with only 3 children scoring 1 *SD* or more below mean. These 3 children were still included as their data do not change the results, and their inclusion increased the power of the analyses. The British Picture Vocabulary Scale 2 (BPVS2; Dunn et al., 1997) was used to assess the children's receptive English vocabulary knowledge. This standardized test provides norms based on monolingual English children in UK aged 3–15, giving some objective measure of the children's proficiency in English. For English, the trilinguals scored on average 52.5 out of a total of 168 items correct (there were more items in BPVS than CRVT as the former can be used for older children), and their performance is more variable (age equivalent according to BPVS: *M*_age_ = 5;2, *SD* = 0;8, *Range* = 3;8–6;5). For Mandarin, we used a receptive vocabulary test we have developed (Chan et al., 2014) that assesses comprehension of 106 words from 14 semantic categories that are chosen based on the early vocabulary inventory of Mandarin-speaking children in Beijing (Hao et al., 2008). As expected, the trilinguals scored significantly lower than the monolingual age-matched comparison group in their Mandarin vocabulary scores [*t*_(22)_ = −5.9, *p* < 0.000, *d* = 1.80]. In fact, these 5- to 6-year-old trilinguals' performed even worse than the 3-year-old monolingual Mandarin group (*N* = 49; aged 2;11–3;05) in the normed sample of the Mandarin receptive vocabulary test (percentage accuracy: trilinguals: *M* = 0.82, *SD* = 0.09; monolinguals: *M* = 0.93, *SD* = 0.036). Tables 2, 3 show the children's performance on the vocabulary tests.

**Table 2:** Vocabulary Scores of the Trilingual Group (chronological age: *M* = 5;8, *SD* = 0;2).

	**Cantonese vocabulary**		**English vocabulary**		**Mandarin vocabulary**	
	***M (SD)***	**Range**	***M (SD)***	**Range**	***M (SD)***	**Range**
**TRILINGUAL**						
Raw score	60 (3)	53–64	52.5 (6.2)	39–63	87.4 (9.99)	67–99
Percentage (%)	92 (5)	82–98	31 (4)	23–38	82 (9)	63–93
Age equivalent	5;8 (0;4)	5;0–6;1	5;2 (0;8)	3;8–6;5	Scores lower than 3-year-old monolingual Mandarin children	

**Table 3:** Vocabulary Scores of the Monolingual Mandarin Comparison Group (chronological age: *M* = 5;11, *SD* = 0;2).

	**Mandarin vocabulary**	
	***M(SD)***	***Range***
**MONOLINGUAL**		
Raw score	101.3 (3.3)	93–106
Percentage (%)	96 (3)	88–100

### Materials and procedure

All children were tested individually by a female experimenter in a quiet room in their school. All children were tested by a native speaker of the respective language. The trilingual children were tested in three sessions, one for each language (vocabulary test first, and then RC test), with the sequence of the languages tested counterbalanced between children.

#### Test of vocabulary knowledge

Test administration followed the standardized test instructions for the CRVT (Cheung et al., 1997), the BPVS2 (Dunn et al., 1997), and the Mandarin receptive vocabulary test (Chan et al., 2014). For all the three vocabulary tests, children were presented with 4 pictures showing the target word and 3 distractor pictures, and were asked to point to the picture that matched a spoken word.

We did not test the monolingual Cantonese children with CRVT, because their CRVT scores were not needed given the following reasons. First, before being confirmed to be able to take part in the study, the monolinguals had been screened by a speech therapist to ensure that they did not present any noticeable speech and language delays in their L1 Cantonese at the time of testing. Second, we intended to match the trilinguals and the Cantonese monolinguals only on their chronological age but not on language proficiency, as the time required for running a full standardized language assessment could not be accommodated by the school. In addition, matching the two groups only on the basis of receptive vocabulary measures to claim for language-matched status is not unproblematic. Third, obtaining the monolinguals' CRVT scores or not would not affect the main pattern of the current findings and their interpretations (see section Discussion for further elaborations).

#### Test of RC comprehension

**Figure 3:**
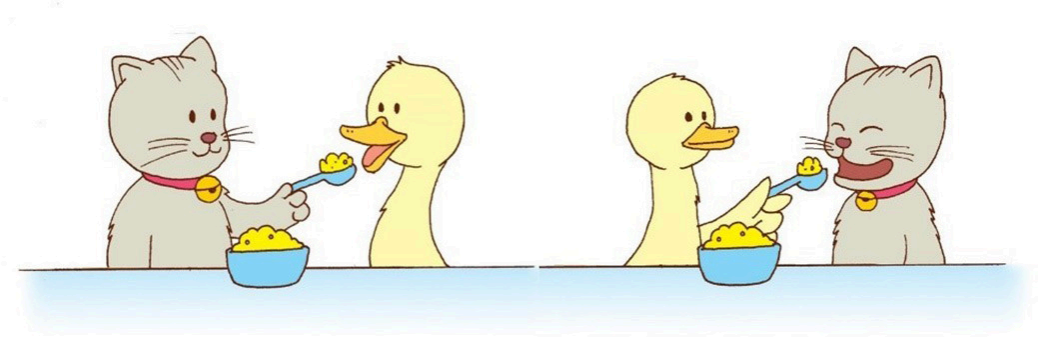
Sample picture pair.

We used the sentence interpretation pointing method and its materials established in Kidd et al. (2015), described briefly below (see Kidd et al., 2015 for details). Children were shown pairs of pictures on a computer screen. Within each pair, both pictures showed the same causative event between two animals and differed only in which animal was the agent and the patient of the action e.g., one picture showed a cat feeding a duck and the other a duck feeding a cat, see Figure 3). Children heard test sentences such as *Where's the duck that is feeding the cat? Find it!* (subject-relative) or *Where's the duck that the cat is feeding? Find it!* (object-relative), and were asked to point to the animal described by the experimenter. Each child received 8 Subject(Agent)- RC test sentences and 8 Object(Patient)- RC test sentences as stimuli for the language being tested, with length and animacy controlled. Table 4 shows examples of the sentence stimuli in the three languages.

**Table 4:** Examples of test sentences in the three languages.

**Sentence type**	**Example**						
Sub-Eng	Where's the cat that is feeding the duck?						
Sub-Can	錫緊	公雞	嗰隻	老鼠	喺	邊度	呀?
	kiss-PROG	chicken	that-CL	mouse	is	where	SFP?
	“Where's the mouse that is kissing the chicken?”						
Sub-Man	抱	小豬	的	小狗	在	哪	?
	hug	piggy	de	doggy	is	where	?
	“Where's the dog that is hugging the pig?”						
Obj-Eng	Where's the horse that the pig is hugging?						
Obj-Man	羊仔	推緊	嗰隻	兔仔	喺	邊度	呀?
	sheep	push-PROG	that-CL	rabbit	is	where	SFP?
	“Where's the rabbit that the sheep is pushing?”						
Obj-Man	白馬	餵	的	老虎	在	哪	?
	white.horse	feed	de	tiger	is	where	?
	“Where's the tiger that the horse is feeding?”						

#### Data coding

Children's responses were coded into four categories: (i) Correct, (ii) Head error: when children pointed to the correct picture but the incorrect animal (e.g., pointing to the cat in the correct picture for the test sentence *Where's the duck that the cat is feeding?*) (iii) Reversal error: when children pointed to the correct token of the head referent in the incorrect picture (e.g., pointing to the picture where the duck is the agent for the test sentence *Where's the duck that the cat is feeding?*), and (iv) Other error: when children pointed to the incorrect animal in the incorrect picture (e.g., pointing to the cat in the incorrect picture for the test sentence *Where's the duck that the cat is feeding?*). The first author coded all the children's responses. One research assistant from each language coded at least 20 percent of the data (at least 10 children from each language) for inter-rater reliability. Inter-rater reliability was close to 100% agreement in all cases.

## Results

**Figure 4:**
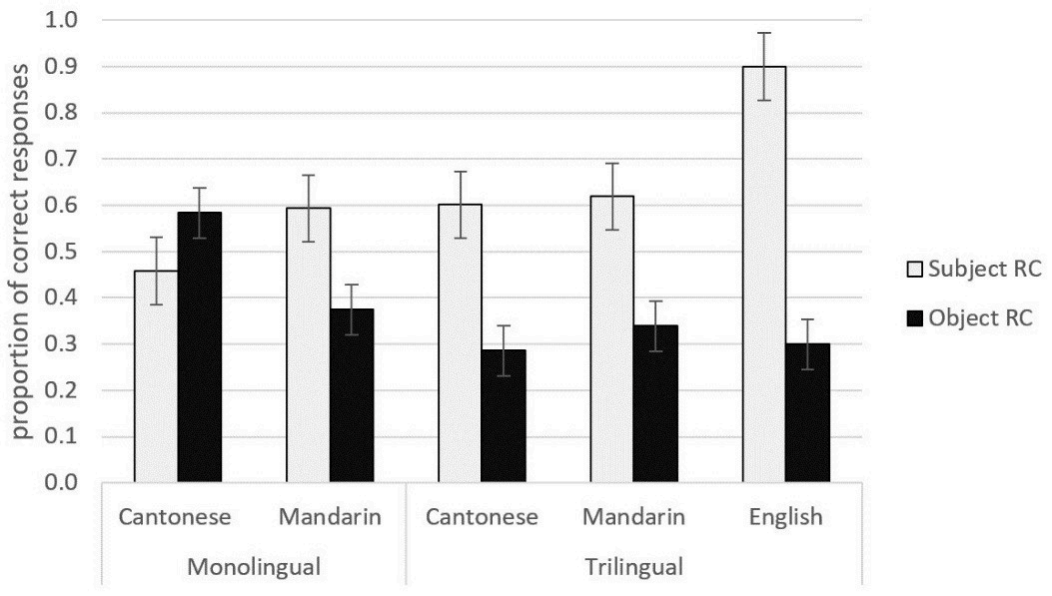
Mean correct performance (and SEs) for trilingual and monolingual children on subject- and object-RCs.

Figure 4 shows the trilingual and monolingual groups' performance on the subject vs. object RCs in Cantonese and Mandarin, and the trilinguals' performance on the English subject- and object- RCs. The trilingual children comprehended subject RCs better than the object RCs for all the three languages (Cantonese: *M*_subjRC_ = 0.60, *M*_objRC_ = 0.29; Mandarin: *M*_subjRC_ = 0.62, *M*_objRC_ = 0.34; English: *M*_subjRC_ = 0.91, *M*_objRC_ = 0.30). The monolingual Mandarin children also comprehended subject RCs better than the object RCs (*M*_subjRC_ = 0.59, *M*_objRC_ = 0.38). In contrast, the monolingual Cantonese children found object RCs easier to comprehend than subject RCs (*M*_subjRC_ = 0.46, *M*_objRC_ = 0.58). In the following sections, we used the R package lme4 (Bates and Maechler, 2010) in R (version 3.3.1, R Core Development Team, 2016) to fit generalized linear mixed models (Jaeger, 2008). The final model was chosen based on significance of fixed effects and random effects. Only significant terms were included.

### Overall analysis

The monolingual and trilingual children's correct responses in Cantonese and Mandarin were analyzed first. The data were analyzed using Generalized Linear Mixed Models (GLMM). This analysis was to test whether there is a significant interaction between Group and Extraction. The fixed effects were Group (trilingual vs. monolingual), Extraction (subject vs. object) and their interaction. Random effects for participants were included to model variation among participants (random intercepts), and by-participant random slopes were also included if significant. Random slopes for the variable of Extraction contributed to model fit significantly and were included in the model. There was a significant Group × Extraction interaction (β = 2.1, *z* = 2.32, *p* = 0.02). This interaction was therefore further scrutinized by analyzing the trilingual vs. monolingual Cantonese groups and the trilingual vs. monolingual Mandarin groups separately using the same analysis strategy.

### Trilingual vs. monolingual Cantonese

Similar to overall analysis in section Overall Analysis, we used GLMM. The analysis was to test whether Group (trilingual vs. monolingual Cantonese), Extraction (subject vs. object) and their interaction significantly contributed to the responses. The fixed effects were Group (trilingual vs. monolingual), Extraction (subject vs. object) and their interaction. Random effects for participants were included to model variation among participants (random intercepts). Random slopes for the variable of Group contributed to model fit significantly and were included in the model. Models were compared with and without random effects (random intercepts or slopes) by likelihood ratio tests to test the significance of them. The final model only included significant random effects. The significant effects for the final model are shown in Table 5. There were significant effects of Extraction and Group, and a significant Group × Extraction interaction. *Post-hoc* analyses that analyzed each extraction type separately showed that the group difference lay crucially in object but not subject RCs. Specifically, the trilinguals comprehended the Cantonese object RC sentences significantly worse than the monolinguals (β = −2.6, *z* = −2.48, *p* = 0.01). When comprehending Cantonese subject RC sentences, the trilinguals performed slightly better than the monolinguals, though the difference was not significant (β = 0.87, *z* = 1.6, *p* = 0.12, *n.s*.).

**Table 5:** Significant terms in final model for analysis of RC Comprehension in Trilingual vs. Monolingual Cantonese.

	**β**	***SE***	***z***	***p***
Intercept	0.392	0.251	1.562	0.118
Extraction	−0.600	0.225	−2.665	0.008^**^^a^
Group	−1.30	0.318	−4.086	<0.001^***^^a^
Group × Extraction	2.499	0.271	9.222	<0.001^***^^a^

a *log likelihood = −829.7, Number of observations = 1,391*,

*** *p < 0.001*,

** *p < 0.01*,

* *p < 0.05*.

### Trilingual vs. monolingual Mandarin

Similar analysis was conducted to test whether Group (trilingual vs. monolingual Mandarin), Extraction (subject vs. object) and their interaction significantly contributed to the responses. In the GLMM we fit, the fixed effects were Group (trilingual vs. monolingual), Extraction (subject vs. object) and their interaction. Random effects for participants were included to model variation among participants (random intercepts). Random slopes for the variable of Extraction contributed to model fit significantly and were included in the model. The significant effects for the final model are shown in Table 6. The only significant effect was that of Extraction, indicating that children comprehended subject RCs better than object RCs in general. Crucially, there was no significant effect of Group and Group did not interact with Extraction, showing that the trilinguals and monolinguals were performing similarly when comprehending Mandarin RCs. This result is interesting because the trilinguals showed similar performance to their age-matched monolingual peers in Mandarin, despite Mandarin being their third and weaker language due to limited exposure (recall that the trilinguals scored significantly lower than the monolinguals in their receptive Mandarin vocabulary, and these trilinguals' receptive Mandarin vocabulary scores were even lower than the 3-year-old monolingual Mandarin group in the normed sample of the vocabulary test). In addition, as Figure 4 shows, the trilinguals displayed strikingly similar performance profiles when comprehending subject and object RCs in their L1 Cantonese and L3 Mandarin, suggesting that positive transfer from Cantonese to Mandarin is implicated. We will return to this point in section Positive Transfer from L1 Cantonese to L3 Mandarin.

**Table 6:** Significant terms in final model for analysis of RC comprehension in Trilingual vs. Monolingual Mandarin.

	**β**	***SE***	***z***	***p***
Intercept	−0.904	0.492	−1.838	0.066
Extraction	1.422	0.657	2.166	0.030^*^^b^
Group	−0.355	0.686	−0.518	0.605
Group: extraction	0.974	0.924	1.054	0.292

b *log likelihood = –728.1, Number of observations = 1,391*,

*** *p < 0.001*,

** *p < 0.01*,

* *p < 0.05*.

Next, we further analyzed data from each group (monolingual Cantonese children, monolingual Mandarin children, and trilingual children) separately.

### Monolingual Cantonese

For monolingual Cantonese children, we tested whether Extraction was significant. In the GLMM, the fixed effect was Extraction (subject vs. object), and the random effects for participants were included to model variation among participants (random intercepts). Random slopes for the variable of Extraction contributed to model fit significantly and were included in the model. Analyses of the monolingual Cantonese data revealed a non-significant effect for Extraction (β = −0.83, *z* = −0.98, *p* = 0.3, *n.s*.), indicating that although the monolingual Cantonese children found object RCs easier to comprehend than subject RCs as shown in Figure 4, the difference was not significant. This slight object advantage is consistent with past comprehension studies on monolingual Cantonese-speaking children's processing of classifier RCs using the same pointing method (Chan et al., 2011; Kidd et al., 2015) as well as using a referent selection eye-tracking task to yield online processing data (Chan et al., 2017).

### Monolingual Mandarin

For monolingual Mandarin children, we tested whether Extraction and Mandarin vocabulary scores were significant. Extraction and Mandarin vocabulary scores were entered as fixed effects. Random slopes for Extraction contributed significantly and were included in the model. There was a significant effect for Extraction (β = 3.16, *z* = 1.98, *p* = 0.048), meaning that the monolingual Mandarin children comprehended subject RCs significantly better than the object RCs, as shown in Figure 4. This subject advantage is consistent with recent experimental findings on monolingual Mandarin-speaking children's processing of RCs (Hsu, 2014; Hu et al., 2015a,b). There was no significant effect for Mandarin vocabulary, likely due to our monolingual children scoring close-to-ceiling in the vocabulary test.

### Trilingual data

We tested whether Extraction was significant for the trilingual children, and whether Mandarin, Cantonese and English vocabulary scores significantly predicted these trilinguals' RC performance. Analyses of the trilingual data revealed a significant effect for Extraction (β = 2.36, *z* = 4.84, *p* < 0.001). Random intercepts and slopes of the variables Extraction, Mandarin Vocabulary and English Vocabulary were significant and were included. There was a marginally significant effect for Mandarin vocabulary as a predictor of the trilinguals' Mandarin RC performance: χ^2^(1) = 3.09, *p* = 0.079 and a marginal significance for Cantonese vocabulary as a predictor of the trilinguals' Cantonese RC performance: Cantonese χ^2^(1) = 3.62, *p* = 0.057), showing that, unsurprisingly, children's RC comprehension in a language improved as their vocabulary scores in the target language increased. There was no significant or a marginally significant effect for English Vocabulary though. We then examined the trilinguals' performance in each language. Random slopes for Extraction contributed significantly and were included in each model for each language. There was a significant effect for Extraction in each language, indicating a significant advantage for subject over object RCs in the trilinguals' L1 Cantonese as well as their L2 English and L3 Mandarin, as shown in Figure 4 (Cantonese: β = 2.53, *z* = 2.39, *p* = 0.017; Mandarin: β = 1.86, *z* = 2.66, *p* = 0.0078; English: β = 4.87, *z* = 5.19, *p* < 0.001).

### Positive transfer from L1 cantonese to L3 Mandarin

In order to further address the likelihood of positive transfer from L1 Cantonese to L3 Mandarin in these trilingual children, we carried out the following analyses. First, we tested whether there were any differences in the proportion of correct responses between the trilinguals' Cantonese vs. Mandarin by fitting mixed effects logistic regression models. We fit two models for a comparison to test a fixed effect of language. The first model only modeled by-participant random intercepts and treats the trilingual's Cantonese and Mandarin as having the same proportion of correct responses. The second model added the fixed effect of language, treating the trilingual's Cantonese and Mandarin as having different proportion of correct responses. A likelihood ratio test was employed to compare the goodness of fit of two models, and test whether there are significant differences between the two models. If there are significant differences, it means that the trilingual's Cantonese and Mandarin have different proportion of correct responses. A likelihood ratio test showed that there was no significant difference between the two models [χ^2^(1) = 0.93, *p* = 0.33], suggesting that the trilinguals' performance in comprehending Cantonese vs. Mandarin RC sentences was similar. Crucially, we also applied the same procedure to test the proportion of correct responses between the trilinguals' Cantonese vs. English in the RC comprehension tasks, but there were significant differences [χ^2^(1) = 18.33, *p* < 0.001], meaning that the trilingual's performance in comprehending Cantonese vs. English RC sentences was different. As Figure 4 shows, there were more correct responses in English than in Cantonese, especially in the subject RC condition. Similarly, there were significant differences in the proportion of correct responses between the trilinguals' Mandarin vs. English in the RC comprehension tasks [χ^2^(1) = 11.7, *p* < 0.001].

Second, to investigate whether the trilinguals' L1 Cantonese RC performance can significantly predict L3 Mandarin RC performance, we fitted a generalized linear mixed effects model, where the response was their binary response in comprehending Mandarin RCs, and the fixed effect was their response in comprehending Cantonese RCs. By-participant random slopes were also included due to significance. Results showed that the trilinguals' Cantonese RC correct performance did significantly contribute to their Mandarin RC correct performance (β = 2.89, *z* = 7.3, *p* < 0.001). Third, additionally, a linear model was fitted with the trilinguals' Mandarin RC scores (the sum of a child's subject and object RC correct responses in the Mandarin RC task) as the responses and their Cantonese RC scores (the sum of a child's subject and object RC correct responses in the Cantonese RC task) as a covariate. The result showed that the trilinguals' L1 Cantonese RC scores positively predicted their L3 Mandarin RC scores (β = 0.59, *t* = 2.26, *p* = 0.036), and this effect remained even after adding Mandarin vocabulary as a covariate. Follow up analyses that analyzed each extraction type separately showed that the trilinguals' L1 Cantonese subject RC scores positively predicted their L3 Mandarin subject RC scores and the result was highly significant (β = 0.78, *t* = 5.81, *p* < 0.001), while the trilinguals' Cantonese object RC scores did not predict their Mandarin object RC scores (β = 0.32, *t* = 1.54, *p* = 0.14, *n.s*.). Importantly, the same analysis strategies were used to examine whether the trilinguals' L1 Cantonese RC scores also predicted their L2 English RC scores, in terms of their combined (subject plus object RC) scores as well as their separate scores for each extraction type, but their L1 Cantonese RC scores did not predict their L2 English RC scores in all these analyses (all *p* > 0.1).

In addition, we used the same analysis strategies to examine whether the trilinguals' L3 Mandarin RC performance also predicted their L1 Cantonese RC performance, in terms of their combined (subject plus object RC) scores as well as their separate scores for each extraction type. First, we fitted a generalized linear mixed effects model, where the response was their binary response in comprehending Cantonese RCs, and the fixed effect was their response in comprehending Mandarin RCs. By-participant random slopes were also included due to significance. Results showed that the trilinguals' Mandarin RC correct performance did significantly contribute to their Cantonese RC correct performance (β = 2.75, *z* = 7.86, *p* < 0.001). Moreover, a linear model was fitted with the trilinguals' Cantonese RC scores (the sum of a child's subject and object RC correct responses in the Cantonese RC task) as the responses and their Mandarin RC scores (the sum of a child's subject and object RC correct responses in the Mandarin RC task) as a covariate. The result showed that the trilinguals' L3 Mandarin RC scores significantly positively predicted their L1 Cantonese RC scores (β = 0.36, *t* = 2.26, *p* = 0.035). Follow up analyses that analyzed each extraction type separately showed that the trilinguals' Mandarin subject RC scores positively predicted their Cantonese subject RC scores and the result was highly significant (β = 0.82, *t* = 5.81, *p* < 0.001), but the trilinguals' Mandarin object RC scores did not predict their Cantonese object RC scores (β = 0.34, *t* = 1.54, *p* = 0.14, *n.s*.).

To summarize, despite showing similar profiles in comprehending subject RCs better than object RCs in all the three languages (see Figure 4), the trilingual children's L1 Cantonese RC scores positively predicted only their L3 Mandarin RC scores but not their L2 English RC scores. In particular, their L1 Cantonese subject RC correct performance strongly and positively predicted their L3 Mandarin subject RC correct performance suggesting positive influence from L1 Cantonese to L3 Mandarin, given the structural parallels between Cantonese and Mandarin RCs as a transparent basis for positive transfer. Interestingly, their L3 Mandarin subject RC correct performance also strongly and positively predicted their L1 Cantonese subject RC correct performance, suggesting that the Cantonese and Mandarin subject RCs share the same representation in these trilinguals. On the other hand, it is also interesting to note that these trilinguals' L1 Cantonese object RC correct performance did not predict their L3 Mandarin object RC correct performance despite the structural overlaps, nor did their L3 Mandarin object RC performance predict their L1 Cantonese object RC performance. This finding is consistent with the idea that children were analyzing the Cantonese object RCs and the Mandarin object RCs differently (which also accords with the linguistic differences between Cantonese and Mandarin object RCs, see section Chinese Relative Clause Processing and Cross-Linguistic Influences in Multilingual Acquisition) and that Cantonese object RCs but not (or to a lesser extent) Mandarin object RCs were subject to cross-linguistic influence from English in these trilinguals.

### Error analyses

**Figure 5:**
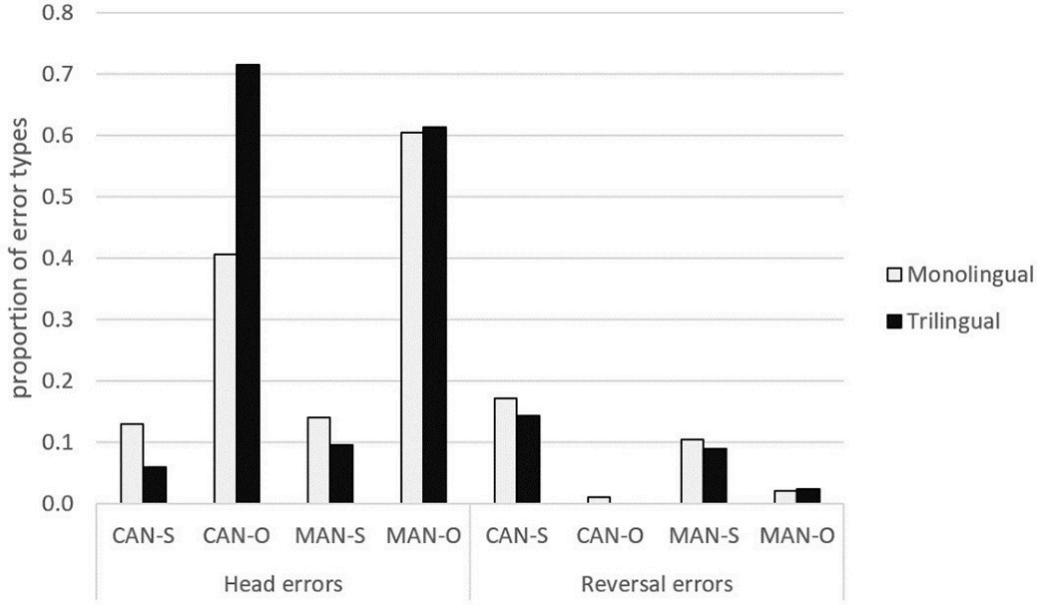
Distribution of error types for monolingual and trilingual groups for subject and object RCs.

We now turn to analyses of the error responses. Children made three error types: (i) head errors, (ii) reversal errors, and (iii) “other” errors. Figure 5 shows the monolingual and trilingual children's average error percentage when comprehending subject and object RCs in Cantonese and Mandarin for each error type.

As in Kidd et al. (2015), only the head errors and reversal errors were analyzed, because, unlike the “other” errors, the processing strategies children use when making these two error types are readily interpretable. Since language is nested under the trilinguals (Cantonese, English, and Mandarin) but not the monolinguals, we compared the trilingual vs. monolingual Cantonese groups and the trilingual vs. monolingual Mandarin groups separately using the same analysis strategy. In addition, their head and reversal error responses were analyzed separately.

#### Head errors

##### Trilingual vs. monolingual Cantonese

We tested whether Extraction, Group (trilingual vs. monolingual Cantonese) and their interaction significantly contributed to head errors. We fitted a linear mixed effects model with the head error responses in trilingual Cantonese and monolingual Cantonese as the response. The fixed effects include Extraction, Group, and their interaction, and the significant random effects by subjects were also included in the model. By likelihood ratio tests, there was a significant effect of Extraction [χ^2^(1) = 36.98, *p* < 0.001] and a significant Group × Extraction interaction [χ^2^(1) = 7.5, *p* = 0.006]. *Post-hoc* analyses that fit a linear regression model to analyze each extraction type separately showed that the group difference lay crucially in object but not subject RCs. Specifically, when comprehending Cantonese object RCs, the trilinguals made significantly more head errors than the monolinguals even though Cantonese is the first language for both groups [*t*_(44)_ = 2.44, *p* = 0.02]. When comprehending Cantonese subject RC sentences, the trilinguals and monolinguals did not exhibit a group difference [*t*_(42)_ = −1.35, *p* = 0.18, *n.s*.].

##### Trilingual vs. monolingual Mandarin

Similarly, we tested whether Extraction, Group (trilingual vs. monolingual Mandarin) and their interaction significantly contributed to head errors. We fitted a linear mixed effects model with the head error responses in trilingual Mandarin and monolingual Mandarin as the response. The fixed effects include Extraction, Group, and their interaction, and the significant random effects by subjects were also included in the model. By likelihood ratio tests, the only significant effect was Extraction [χ^2^(1) = 41.131, *p* < 0.001], indicating that children made head errors significantly more often when comprehending Mandarin object RCs than Mandarin subject RCs. There was no significant effect of Group and it did not interact with Extraction, showing that the trilinguals had similar head noun error rate compared to their age-matched monolinguals when comprehending Mandarin RCs, a finding that is also consistent with comparing the two groups based on their correct responses.

#### Reversal errors

##### Trilingual vs. monolingual Cantonese

We tested whether Extraction, Group (trilingual vs. monolingual Cantonese) and their interaction significantly contributed to reversal errors. We used the same analysis strategy fitting a linear mixed effects model including random effects for subjects with the reversal error responses in trilingual Cantonese vs. monolingual Cantonese as the response, and Extraction, Group, and their interaction as fixed factors. By likelihood ratio tests, the only significant effect was Extraction [χ^2^(1) 22.04, *p* < 0.001], indicating that children made reversal errors significantly more often when comprehending Cantonese subject RCs (non-canonical VOS) than Cantonese object RCs (canonical SVO) in general. There was no significant effect of Group and it did not interact with Extraction, showing that the trilinguals and monolinguals were similar in terms of their tendency to make reversal errors when comprehending Cantonese RCs.

##### Trilingual vs. monolingual Mandarin

We tested whether Extraction, Group (trilingual vs. monolingual Mandarin) and their interaction significantly contributed to reversal errors. Likewise, we used the same analysis strategy to compare reversal error responses in trilingual Mandarin vs. monolingual Mandarin. The major results are similar to those comparing reversal errors in trilingual vs. monolingual Cantonese. The only significant effect was Extraction [Ext χ^2^(1) 5.19, *p* = 0.02], indicating that children made reversal errors significantly more often when comprehending Mandarin subject RCs (non-canonical VOS) than Mandarin object RCs (canonical SVO) in general. There was no significant effect of Group and it did not interact with Extraction, showing that the trilinguals and monolinguals were similar in terms of their tendency to make reversal errors when comprehending Mandarin RCs.

### Negative transfer from L2 English to L1 Cantonese

To summarize, our error analyses revealed a crucial difference between the trilinguals and their age matched monolinguals when they comprehended Cantonese object RCs: the trilinguals made significantly more head errors than the monolinguals even though Cantonese is the first language for both groups. That is, the trilinguals were more likely to erroneously choose the subject of the RC as the head noun, choosing “mouse” instead of “chicken” as the head noun in (5) repeated as (14) below.

**Cantonese object classifier RC (head-final)**

(14) [**_RC_**  老鼠          錫___ _j_] [_headnoun_   嗰    隻       公雞_j_]
               lou5syu2   sek3                    go2  zek3   gung1gai1
               mouse       kiss                     that  CL     chicken
               “The chicken that the mouse kisses”

We suggest that this group difference can be attributed to the trilinguals' knowledge of English, specifically these head errors in Cantonese could result from applying an English-based parsing strategy to the Cantonese object classifier RC stimuli. We will elaborate this argument further in the Discussion section.

## Discussion

We have presented data involving the acquisition of two Chinese languages in a group of trilingual children who are also intensively exposed to English at school. The children from this study are acquiring the two Chinese languages under different exposure conditions, Cantonese as first language, Mandarin as their third language, under the heavy influence of English. We examined how these children's comprehension of subject and object RCs in the two Chinese languages is related to their knowledge of Cantonese, English and Mandarin. The results showed effects of both positive transfer and negative transfer across the three languages, showing bi-directional influence between the first and second/third languages. In particular, positive transfer from L1 Cantonese to L3 Mandarin allowed the trilingual children to comprehend Mandarin RCs above the level that would be expected based on their limited input. In contrast, negative transfer from L2 English to L1 Cantonese resulted in trilingual children having more difficulties in comprehending Cantonese object classifier RCs relative to their monolingual age peers.

Our hypotheses were that these trilinguals would experience facilitation in comprehending RCs in their L3 Mandarin; but would experience more difficulty in processing object classifier RCs in their L1 Cantonese relative to their monolingual peers. Our hypotheses are supported. In Mandarin, the trilinguals performed on a par with their monolingual age matched peers in comprehending complex sentences such as RCs, although Mandarin is their third and weaker language due to limited exposure. Recall these 5- to 6- year-old trilinguals scored lower than even the 3-year-old monolingual Mandarin children in terms of receptive vocabulary competence. In addition, their Cantonese RC performance and Mandarin RC performance were strikingly similar (see Figure 4), leading us to argue that positive transfer from their first language Cantonese to their third language Mandarin is implicated. Our argument for positive forward transfer is further substantiated by showing that the trilinguals' L1 Cantonese RC performance uniquely positively predicts their L3 Mandarin RC performance, in particular for subject RCs. By contrast, their L1 Cantonese RC performance did not predict their L2 English RC performance, although the trilinguals exhibited a subject advantage across all the three languages.

In Cantonese, although having been extensively exposed to it from family and community since birth and their Cantonese receptive vocabulary scores are comparable to their age peers, the trilinguals performed significantly worse than the age-matched monolingual Cantonese group in comprehending Cantonese object classifier RCs because the trilinguals made significantly more head errors when parsing this construction. In these errors, the subject of the RC was mistakenly interpreted as the semantic head of a relative clause. These head errors could be a manifestation of negative influence from English, resulting from the trilinguals applying an English-based parsing strategy to the Cantonese object RC stimuli. Recall that Cantonese object RCs overlap with English head-initial subject RCs, in addition to overlapping with SVO transitive main clauses in all three languages. The trilinguals may have misparsed the Cantonese object RC stimuli using the English-based “head initial” analysis, erroneously taking the subject and the first noun (mouse), instead of the object and second noun chicken, as the semantic head of the relative clause, as shown in (15). A number of mechanisms may be implicated in the transfer of the English head-initial analysis. First, as described in the section Introduction, Cantonese object classifier RCs allow for an alternative internally headed analysis and this is a typological feature unique to Cantonese (see Chan et al., 2011 for more details). One possibility is thus that the head error arises from taking the subject to be the semantic head of an internally-headed relative clause.

(15) [_**RC** headnoun_    老鼠           錫       緊               嗰     隻
                             **lou5syu2**   sek3   gan2           go2   zek3
                             **mouse**       kiss    PROG       that   CL
                             公雞]          喺       邊    度       呀?
                             gung1gai1  hai2    bin1dou6   aa3
                             chicken      is       where        SFP
                             (meaning: “where's the mouse that is kissing the chicken?”)

A second factor is that mis-parsing may be facilitated by the presence of the progressive aspect marker (PROG) *gan2* in the Cantonese stimuli which corresponds closely to the English suffix –*ing* [see (15) and Table 4 for examples of test sentences]. Given this correspondence, it is possible that the trilinguals misparsed the Cantonese object RC stimuli similar to an English reduced subject RC [the mouse (that's) kissing the chicken], resulting in the head assignment error. Such English-based effects align with the fact that the trilingual and the monolingual groups differ crucially in terms of their exposure to English. This interpretation of the error pattern predicts that children would make more head errors with Cantonese object RCs as their English dominance increased. We examined whether measures of language dominance or English proficiency would predict children's head noun errors in Cantonese object RCs in these trilinguals but found null results on this point. In a relevant study by Kidd et al. (2015, p. 447), however, a dominance effect was attested, as the study reported the main effect of dominance approaching significance in 20 simultaneous Cantonese-English bilinguals (*p* = 0.07), suggesting that the children made fewer head errors as their Cantonese dominance increased. The difference in findings could be due to the fact that the bilingual children in Kidd et al. (2015) lived in an English-speaking environment (Canberra, Australia), and are thus likely to be more English dominant overall. We therefore concur with Kidd et al. (2015)'s suggestion that an important follow-up study would be to test a larger group of multilingual children with a wider array of dominance profiles.

One possibility as an alternative explanation is that these trilinguals were using an immature parsing strategy characteristic of younger monolingual Cantonese language learners. This alternative explanation is unlikely. First, although the trilingual and monolingual groups were not matched on their vocabulary scores, the trilinguals' performance in the L1 Cantonese vocabulary test was comparable to their age matched peers in the normed sample of the test, and the monolingual and trilingual groups in this study were age matched. Second, the trilinguals and monolinguals performed similarly on the Cantonese subject RCs, which for the monolinguals appear to be more difficult than object RCs. Third, even when we attempted to compare the trilinguals' performance profile in Cantonese with that of a younger group of monolingual Cantonese learners from another study reported in Chan et al. (2011), they are also distinctly different. Given that the trilingual group and the monolingual Cantonese group in the current study differ crucially in terms of their English exposure, and that their head errors were consistent with an English-based head-initial analysis, with structural overlaps between languages as a pre-condition, we therefore argue that the trilinguals' higher rate of head noun errors was more likely due to cross-linguistic influence from English.

The new findings complement and extend our previous works in a number of ways. They confirm our observation of cross-linguistic influence in Chinese-English bilingual's acquisition of RCs and extend this observation from simultaneous bilinguals to child second language acquisition. The presence of competing constructions makes SVO head-final object classifier RCs especially vulnerable in multilingual L1 Cantonese. Such vulnerability echoes the vulnerability reported in the L1 Cantonese of a group of simultaneous Cantonese-English bilingual children in Australia (Kidd et al., 2015). Our findings provide further evidence that head noun assignment in object classifier RCs is especially vulnerable to errors in multilingual Cantonese children under intensive exposure to English, even when they have been exposed to Cantonese as first language from birth. Our results therefore extend Kidd et al. (2015)'s observation of negative transfer from English to Cantonese attested in a group of simultaneous bilinguals to another group of multilingual children who are also acquiring Cantonese under intensive exposure to English. Investigation of vulnerable linguistic domains in different multilingual child populations in relation to language exposure conditions and the language pair(s) involved can inform researchers about when and where cross-linguistic influence occurs in bilingual or multilingual development on one hand; and inform practitioners about when and where focused remediation may be considered on the other hand.

An additional empirical dimension offered by this study involves interactions between two Chinese languages (Cantonese and Mandarin) in multilingual child development. Specifically, in a trilingual Cantonese-English-Mandarin acquisition context, this study documents positive transfer from Cantonese to Mandarin between the trilinguals' first and third languages. Given that Mandarin is gaining prestige as a lingua franca among Chinese people in China, Hong Kong, Taiwan, Singapore and overseas communities, it is increasingly common for Chinese children to acquire one Chinese language as their home and first language, while also acquiring Mandarin simultaneously or successively from the community and/or school. These children develop some form of bilingualism involving two Chinese languages under different exposure conditions. The finding regarding positive transfer between the two Chinese languages also bears on the education of bilingual and trilingual children. Here we see the merit of children's L1 Cantonese benefiting the processing and acquisition of comparable structures in their L3 Mandarin despite limited exposure to Mandarin. We take this beneficial effect as a good reason to promote proficiency of Cantonese as heritage language for these trilingual children in their school and family education.

On the other hand, we also see interesting selective evidence of positive transfer from Cantonese to Mandarin in our trilingual children specific to one relative clause structure (subject relative) but not the other (object relative) that matches well with the similarities and differences between Cantonese and Mandarin relative clauses. Grammatical differences between Chinese languages and their implications for language acquisition have not received much attention so far. Investigating the acquisition of Chinese languages in bilingual/multilingual development requires recognizing that there are varieties of Chinese and considering the diversity in the specific properties of the target Chinese languages as an important linguistic factor in specifying where domains of facilitation or vulnerability may lie. For instance, we predict that positive transfer would not work for specific domains of grammar, such as acquiring the word order of double object datives in which Cantonese and Mandarin differ (Chan, 2010).

The current findings relate to theoretical perspectives in multilingual language acquisition and processing, especially with respect to cross-linguistic influences, in a number of ways. The positive transfer from L1 to the weaker L3 observed at such a young age is theoretically interesting from the perspective of psychotypology (the perception of linguistic distance). In the case of child language learners, age is associated with cognitive and metalinguistic development, and cognitive and metalinguistic development could in turn be related to psychotypology: in general, one would expect that older children who have developed higher metalinguistic awareness may have a more accurate perception of linguistic distance. To the extent that psychotypology is possibly involved in the current group of 5- to 6- year-old trilinguals, it is impressive to observe young children having a perception of linguistic proximity between the two Chinese languages that could trigger forward positive transfer in processing certain similar syntactic structures. Taken together, then, in addition to the presence of structural parallels as a pre-condition for cross-linguistic transfer, positive transfer from L1 Cantonese to L3 Mandarin could be jointly driven by factors such as actual and perceived language distance (given the typological proximity between Cantonese and Mandarin) and language dominance (given that the trilinguals' Cantonese is more dominant than their Mandarin). Furthermore, recall the trilinguals' L1 Cantonese subject RC correct performance strongly and positively predicted their L3 Mandarin subject RC correct performance, and vice versa. This finding is also theoretically interesting because it constitutes suggestive evidence for shared syntactic representations between Cantonese and Mandarin in these young trilinguals and co-activation of their two typologically close languages during processing. The result is also consistent with psycholinguistic theories for bilinguals that posit shared syntactic representations between languages in instances of surface structure overlap (e.g., Meijer and Fox Tree, 2003; Hartsuiker and Pickering, 2008). To further test this hypothesis, a follow up study could be to test whether multilingual children acquiring Cantonese and Mandarin show any between-language priming effects between Cantonese and Mandarin (for subject RCs but not object RCs).

The finding regarding directionality of transfer from English to Cantonese is consistent with the prediction derived from Hulk and Müller's hypothesis. Recall that Cantonese object classifier RCs are potentially ambiguous between more than one analysis, and these Cantonese object classifier RCs overlap with subject RCs in English when the two languages are in contact in multilingual acquisition, while English RCs clearly allow only a head-initial analysis. According to Hulk and Müller's hypothesis, it would predict that Cantonese is the language being affected by cross-linguistic influence from English. Reverse transfer from L2 English to L1 Cantonese could therefore be triggered, especially when the children are under intensive exposure to English. The current finding further confirms the idea that if it is the structure in the first language that presents potential ambiguity of analyses, and the overlapping structure in the second language presents no ambiguity of analysis, reverse transfer from L2 to L1 is possible between two typologically divergent languages like Cantonese and English. In fact, such reverse transfer may be more likely to occur at an early age, during which the grammatical system of even the first language is under development for a multilingual child, making it more susceptible to cross-linguistic influence in vulnerable domains where structural ambiguity and competing analyses take time to resolve in the presence of structural overlaps. The current finding demonstrates that Hulk and Müller's hypothesis suffices to provide a unified theoretical perspective to jointly consider cross-linguistic influence across bilingual and trilingual acquisition contexts.

A further remark about the effect of transfer. The structural overlap condition and Hulk and Müller's cross-linguistic influence hypothesis did not make specific predictions regarding whether the transfer is positive or negative. We view positive/negative transfer as an outcome rather than a process. The outcome depends on whether the overlapping analysis would lead to accurate usage/comprehension or errors/non-target forms in the target language. For subject RCs in Cantonese and Mandarin, the overlapping analysis leads to correct interpretation, hence positive transfer. For object classifier RCs in Cantonese, the overlapping analysis leads to incorrect interpretation, hence negative transfer. In the current case of negative transfer, we would like to further elaborate on the overlapping analysis, because the head-initial RC analysis preferred by the trilinguals is not permitted by the grammar of Cantonese. It is relevant to note that the Cantonese monolinguals also made this kind of head errors when comprehending object classifier RCs, although to a significantly lesser degree. This is not surprising in light of Hulk and Müller's cross-linguistic influence hypothesis and their notion of “vulnerable domain”: cross-linguistic influence would occur in domains that are also known to be vulnerable and challenging for monolingual children. We further hypothesize that there is a coalition of “1st noun-as-agent” and “1st noun-as-head noun” processing preferences for young children in general (Bever, 1970; MacWhinney, 1977; Diessel and Tomasello, 2005). This general developmental tendency enables children to have good performance in comprehending subject RCs in languages with head-initial RCs like English and German (Diessel and Tomasello, 2005), but would give rise to developmental errors in head noun assignment for children acquiring head-final RCs, because “2nd N patient-as-head noun” conflicts with “1st N as agent and head noun” general processing preference. This hypothesis is further motivated by observing our published and unpublished data featuring languages like Cantonese, Mandarin and Dong with SVO head-final RCs that such kind of head noun assignment errors are not uncommon even among monolingual children (Yang and Chan, 2014; Kidd et al., 2015; Chan et al., 2017). Following this hypothesis, the overlapping analysis for our trilinguals in the current study would be the head-initial RC analysis, which is uniformly attested in English on one hand, and aligned with young children's general processing preferences on the other hand. This head-initial RC analysis therefore led to head noun assignment errors when the trilinguals comprehended Cantonese object classifier RCs, erroneously choosing the subject of the RC as the head noun. We can further view the mechanism of this negative transfer from a usage-based perspective (Tomasello, 2003; Lieven and Tomasello, 2008). The idea is that these developmental head noun assignment errors might be *more entrenched* in the trilingual children's Cantonese than those in the monolingual children. What is different between the trilingual and monolingual children's linguistic experience is that these trilingual children heard invariant head-initial RC forms in their additional and intensive English input. Apart from structural overlaps between object classifier RCs in Cantonese and simple SVO transitive constructions in Cantonese and English, and subject RCs from English which are also SVO in order, there is also structural overlap between the invariant head-initial RC analysis from English and children's developmental tendency to choose the first mentioned noun phrase as the agent and the head noun of an SVO RC in Cantonese. As such, the tokens of head-initial RC forms in English that the trilingual children heard could have further entrenched these children's developmental tendency, making them increasingly accessible when it comes to syntactic choice in comprehension, leading to higher error frequency.

Limitations of the current study are also highlighted as follows. Trilingual language learners in the early years have three developing systems that can potentially influence each other. This study focused on documenting and accounting for the binary interactions between English and Cantonese (whereby English negatively influenced the parsing of a Cantonese grammatical construction), and the binary interactions between Cantonese and Mandarin (whereby Cantonese positively influenced Mandarin, with a possibility that Cantonese and Mandarin subject RCs have a shared representation in these trilinguals). Directionality of influence from English to Mandarin remains possible, but is difficult to test in the current case, mainly because if English were also influencing the non-dominant Mandarin, Mandarin is likely being jointly influenced by both Cantonese and English, and as such the joint influences cannot be teased apart. This study is therefore unable to investigate all the possible pathways of cross-linguistic influences between the three languages. Despite this, the current study points to an exciting new line of inquiry for future research. A fair amount of works on trilingualism have so far focused on how English as a lingua franca interacts with other languages in the European context (Cenoz and Jessner, 2000). As Mandarin becomes increasingly popular to acquire as a foreign language both for children and adults on a global scale, it will be extremely exciting to study how Mandarin is acquired as a L3 and how it interacts with other languages in a global context. A final remark about language dominance. While we are certain that Mandarin is the trilinguals' weakest language given their limited input (in contrast to Cantonese and English, which both featured prominently in these children's daily input), we are unsure about their relative dominance between Cantonese and English, as we have not used a comparable set of measures to systematically assess and compare these children's proficiency in Cantonese and English. Having only the receptive vocabulary scores from two different tests (CRVT and BPVS) does not allow us to make solid claims about the Cantonese-English dominance profiles of these trilinguals. The extent to which transfer from English to Cantonese is also driven by these trilinguals' dominance in English is unknown at the moment.

## Conclusion

This study is one of the very few studies that address cross-linguistic influences in young sequential trilingual children. We have identified a specific case of bidirectional influence between the first and second/third languages in Cantonese-English-Mandarin trilingual children's comprehension of relative clauses. On the one hand, parallels and overlaps in both form and function provide a transparent basis for positive transfer from L1 Cantonese to L3 Mandarin, instantiating forward positive transfer from L1 to L3. On the other hand, intensive exposure to L2 English and structural overlaps in the languages cause multilingual children to experience more difficulty in processing object classifier RCs in their L1 Cantonese relative to their monolingual peers, instantiating backward negative transfer from L2 English to L1 Cantonese. These bi-directional cross-linguistic influences were attested within a single syntactic domain, demonstrating robust interactions between the linguistic systems of multilingual children. This study demonstrates how cross-linguistic interactions and exposure conditions could jointly influence acquisition outcomes: in this case, processing is facilitated by positive cross-linguistic influence despite limited exposure, and inhibited despite extensive exposure from birth due to negative cross-linguistic influence.

## Ethics statement

This study was carried out in accordance with the recommendations of the Human Subjects Ethics Sub-committee at the Hong Kong Polytechnic University. Ethics approval has been sought (reference number: HSEARS20150128006). Written informed consent was also obtained from the parents of each participant.

## Author contributions

AC designed the experiment and interpreted the data in consultation with SM and VY. AC recruited the participants, supervised native-speaker experimenters in data collection, coded the data, supervised research assistants in data management such as reliability checks, and wrote a first draft of the paper. SC ran the statistical analyses. Subsequently all authors worked on refining and revising the text. All authors approved the final version.

### Conflict of interest statement

The authors declare that the research was conducted in the absence of any commercial or financial relationships that could be construed as a potential conflict of interest.

## Footnotes

^1^We did not use standardized language assessments such as Reynell Developmental Language Scales (Reynell and Huntley, 1987) and HKCOLAS (T'sou et al., 2006). Although they offer more comprehensive information including morphosyntax, they take much longer to run than could be accommodated by the school.
